# (1*E*,2*E*)-1,2-Bis(3-bromo-4-meth­oxy­benzyl­idene)hydrazine

**DOI:** 10.1107/S1600536811016904

**Published:** 2011-05-11

**Authors:** Jerry P. Jasinski, James A. Golen, C. S. Chidan Kumar, B. Narayana, H. S. Yathirajan

**Affiliations:** a Department of Chemistry, Keene State College, 229 Main Street, Keene, NH 03435-2001, USA; b Department of Studies in Chemistry, University of Mysore, Manasagangotri, Mysore 570 006, India; c Department of Studies in Chemistry, Mangalore University, Mangalagangotri 574 199, India

## Abstract

In the title compound, C_16_H_14_Br_2_N_2_O_2_, the dihedral angle between the mean planes of the two benzene rings is 33.4 (2)°. The hydrazine group is twisted slightly, with C—N—N—C and C—C—N—N torsion angles of 167.5 (4) and 177.2 (4)/174.2 (4)°, respectively.

## Related literature

For anti­tubercular behaviour in isonicotinoyl hydrazones, see: Kucukguzel *et al.* (1999 ▶); Rollas *et al.* (2002 ▶). For the coordination chemistry of azine compounds containing both a diamine linkage and an N—N bond, see: Armstrong *et al.* (1998 ▶); Kesslen & Euler (1999 ▶); Kundu *et al.* (2005 ▶); Xu *et al.* (1997 ▶). For related structures, see: Zheng *et al.* (2005 ▶, 2006 ▶); Zheng & Zhao (2006 ▶); Odabaşoğlu *et al.* (2007 ▶).
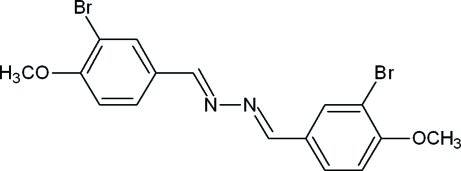


## Experimental

### 

#### Crystal data

C_16_H_14_Br_2_N_2_O_2_
*M* *_r_* = 426.11
Monoclinic, 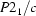

*a* = 10.1354 (8) Å
*b* = 10.550 (1) Å
*c* = 15.6055 (12) Å
β = 96.680 (7)°
*V* = 1657.3 (2) Å^3^
*Z* = 4
Mo *K*α radiation
μ = 4.90 mm^−1^
*T* = 173 K
0.20 × 0.15 × 0.10 mm

#### Data collection

Oxford Xcalibur Eos Gemini diffractometer
Absorption correction: multi-scan (*CrysAlis RED*; Oxford Diffraction, 2010 ▶) *T*_min_ = 0.441, *T*_max_ = 0.640
14411 measured reflections
3941 independent reflections
2537 reflections with *I* > 2σ(*I*)
*R*_int_ = 0.061

#### Refinement

*R*[*F* ^2^ > 2σ(*F* ^2^)] = 0.053
*wR*(*F* ^2^) = 0.120
*S* = 1.09
3941 reflections
201 parameters
H-atom parameters constrained
Δρ_max_ = 0.53 e Å^−3^
Δρ_min_ = −0.64 e Å^−3^

### 

Data collection: *CrysAlis PRO* (Oxford Diffraction, 2010 ▶); cell refinement: *CrysAlis PRO*; data reduction: *CrysAlis RED* (Oxford Diffraction, 2010 ▶); program(s) used to solve structure: *SHELXS97* (Sheldrick, 2008 ▶); program(s) used to refine structure: *SHELXL97* (Sheldrick, 2008 ▶); molecular graphics: *SHELXTL* (Sheldrick, 2008 ▶); software used to prepare material for publication: *SHELXTL*.

## Supplementary Material

Crystal structure: contains datablocks global, I. DOI: 10.1107/S1600536811016904/tk2741sup1.cif

Structure factors: contains datablocks I. DOI: 10.1107/S1600536811016904/tk2741Isup2.hkl

Supplementary material file. DOI: 10.1107/S1600536811016904/tk2741Isup3.cml

Additional supplementary materials:  crystallographic information; 3D view; checkCIF report

## Figures and Tables

**Fig. 1.**
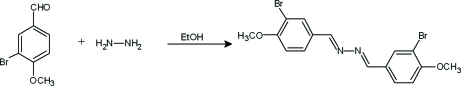
Reaction scheme for the title compound.

**Fig. 2.**
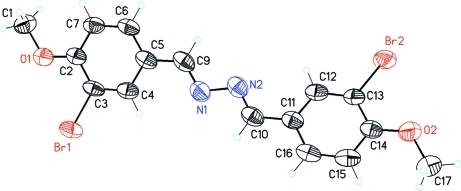
Molecular structure of the title compound showing the atom labeling scheme and 50% probability displacement ellipsoids.

## supplementary crystallographic information

### Comment

Hydrazones are known to possess antimicrobial, anticonvulsant, analgesic, anti-inflammatory, antiplatelet, antitubercular and antitumoral activities. For example, isonicotinoyl hydrazones are antitubercular; 4-fluorobenzoic acid [(5-nitro-2-furyl)methylene]-hydrazide (Rollas *et al.*, 2002) and 2,3,4-pentanetrione-3-[4-[[(5-nitro-2-furyl)methylene]hydrazine] carbonyl]phenyl]hydrazone (Kucukguzel *et al.*, 1999) have antibacterial activity. A number of azine compounds containing both a diamine linkage and an N—N bond have been investigated in terms of their crystallography and coordination chemistry (Kundu *et al.*, 2005; Kesslen & Euler, 1999; Armstrong *et al.*, 1998; Xu *et al.*, 1997). The crystal structures of N,N'-Bis(3-nitrobenzylidene)hydrazine (Zheng *et al.*, 2005), N,N'-Bis(2,6-dichlorobenzylidene)hydrazine (Zheng *et al.*, 2006), N,N'-Bis(9-anthracenylidene)hydrazine (Zheng & Zhao, 2006), 4-Fluorobenzaldehyde [(E)-4-fluorobenzylidene] hydrazone (Odabaşoğlu *et al.*, 2007) have been reported. In view of the importance of hydrazones, the title compound, C_16_H_14_Br_2_N_2_O_2_, (I), is synthesized, Fig. 1, and its crystal structure is reported here.

In (I) the dihedral angle between the mean planes of the two benzene rings is 33.4 (2)° (Fig. 2). The hydrazine group is twisted slightly with C9—N1—N2—C10, N2—N1—C9—C5 and N1—N2—C10—C11 torsion angles of 167.5 (4) and 177.2 (4) and 174.2 (4)°, respectively. The crystal packing is stabilized by weak van der Waals interactions.

### Experimental

A mixture of 3-bromo-4-methoxy benzaldehyde (4.3 g, 0.02 mol) and hydrazine hydrate (0.5 ml, 0.01 mol) in 15 ml of ethanol containing 2 drops of 4 M sulfuric acid was refluxed for about 3 h (Fig. 1). On cooling, the solid separated, was filtered and recrystallized from N,N-dimethylformamide (m.p. 453–455 K).

### Refinement

The parameters of all the H atoms have been constrained within the riding atom approximation. C—H bond lengths were constrained to 0.95 Å for aryl atoms, *U*_iso_(H) = 1.19–1.20*U*_eq_(C_aryl_), and 0.98 Å for methyl atoms, *U*_iso_(H) = 1.49*U*_eq_(C_methyl_).

### Crystal data

**Table:**

C_16_H_14_Br_2_N_2_O_2_	*F*(000) = 840
*M**_r_* = 426.11	*D*_x_ = 1.708 Mg m^−^^3^
Monoclinic, *P*2_1_/*c*	Mo *K*α radiation, λ = 0.71073 Å
Hall symbol: -P 2ybc	Cell parameters from 3889 reflections
*a* = 10.1354 (8) Å	θ = 3.0–32.2°
*b* = 10.550 (1) Å	µ = 4.90 mm^−^^1^
*c* = 15.6055 (12) Å	*T* = 173 K
β = 96.680 (7)°	Block, yellow
*V* = 1657.3 (2) Å^3^	0.20 × 0.15 × 0.10 mm
*Z* = 4	

### Data collection

**Table:**

Oxford Xcalibur Eos Gemini diffractometer	3941 independent reflections
Radiation source: Enhance (Mo) X-ray Source	2537 reflections with *I* > 2σ(*I*)
graphite	*R*_int_ = 0.061
Detector resolution: 16.1500 pixels mm^-1^	θ_max_ = 27.9°, θ_min_ = 3.0°
ω scans	*h* = −13→13
Absorption correction: multi-scan (*CrysAlis RED*; Oxford Diffraction, 2010)	*k* = −13→13
*T*_min_ = 0.441, *T*_max_ = 0.640	*l* = −20→20
14411 measured reflections	

### Refinement

**Table:**

Refinement on *F*^2^	Primary atom site location: structure-invariant direct methods
Least-squares matrix: full	Secondary atom site location: difference Fourier map
*R*[*F*^2^ > 2σ(*F*^2^)] = 0.053	Hydrogen site location: inferred from neighbouring sites
*wR*(*F*^2^) = 0.120	H-atom parameters constrained
*S* = 1.09	*w* = 1/[σ^2^(*F*_o_^2^) + (0.0419*P*)^2^ + 0.0769*P*] where *P* = (*F*_o_^2^ + 2*F*_c_^2^)/3
3941 reflections	(Δ/σ)_max_ = 0.002
201 parameters	Δρ_max_ = 0.53 e Å^−^^3^
0 restraints	Δρ_min_ = −0.64 e Å^−^^3^

### Special details

**Table:**

Geometry. All esds (except the esd in the dihedral angle between two l.s. planes) are estimated using the full covariance matrix. The cell esds are taken into account individually in the estimation of esds in distances, angles and torsion angles; correlations between esds in cell parameters are only used when they are defined by crystal symmetry. An approximate (isotropic) treatment of cell esds is used for estimating esds involving l.s. planes.
Refinement. Refinement of *F*^2^ against ALL reflections. The weighted *R*-factor *wR* and goodness of fit *S* are based on *F*^2^, conventional *R*-factors *R* are based on *F*, with *F* set to zero for negative *F*^2^. The threshold expression of *F*^2^ > σ(*F*^2^) is used only for calculating *R*-factors(gt) *etc*. and is not relevant to the choice of reflections for refinement. *R*-factors based on *F*^2^ are statistically about twice as large as those based on *F*, and *R*-factors based on ALL data will be even larger.

### Fractional atomic coordinates and isotropic or equivalent isotropic displacement parameters (Å^2^)

**Table:**

	*x*	*y*	*z*	*U*_iso_*/*U*_eq_	
Br1	0.58094 (5)	0.79236 (5)	1.06171 (3)	0.06434 (19)	
Br2	0.19292 (5)	0.55086 (6)	0.31176 (3)	0.0745 (2)	
O1	0.7706 (3)	0.5773 (3)	1.09367 (19)	0.0569 (8)	
O2	−0.0712 (3)	0.6690 (3)	0.3128 (2)	0.0651 (9)	
N1	0.3700 (4)	0.6100 (4)	0.7479 (2)	0.0593 (10)	
N2	0.3124 (4)	0.5901 (4)	0.6626 (2)	0.0564 (10)	
C1	0.8682 (5)	0.4829 (5)	1.1222 (3)	0.0741 (15)	
H1B	0.9152	0.5082	1.1781	0.111*	
H1C	0.9319	0.4750	1.0799	0.111*	
H1D	0.8241	0.4013	1.1283	0.111*	
C2	0.6994 (4)	0.5613 (4)	1.0151 (3)	0.0464 (10)	
C3	0.6047 (4)	0.6531 (4)	0.9888 (3)	0.0472 (10)	
C4	0.5291 (4)	0.6453 (4)	0.9104 (3)	0.0490 (10)	
H4A	0.4646	0.7088	0.8938	0.059*	
C5	0.5461 (4)	0.5448 (4)	0.8545 (3)	0.0498 (11)	
C6	0.6410 (4)	0.4535 (4)	0.8803 (3)	0.0531 (11)	
H6A	0.6539	0.3847	0.8429	0.064*	
C7	0.7170 (4)	0.4611 (5)	0.9595 (3)	0.0543 (12)	
H7A	0.7815	0.3977	0.9761	0.065*	
C9	0.4677 (5)	0.5372 (5)	0.7703 (3)	0.0547 (12)	
H9A	0.4905	0.4751	0.7305	0.066*	
C10	0.2020 (5)	0.6449 (5)	0.6464 (3)	0.0553 (11)	
H10A	0.1649	0.6860	0.6922	0.066*	
C11	0.1288 (4)	0.6482 (4)	0.5605 (3)	0.0482 (10)	
C12	0.1842 (4)	0.6029 (4)	0.4883 (3)	0.0502 (11)	
H12A	0.2701	0.5657	0.4953	0.060*	
C13	0.1157 (4)	0.6119 (4)	0.4083 (3)	0.0467 (10)	
C14	−0.0115 (4)	0.6638 (4)	0.3947 (3)	0.0487 (10)	
C15	−0.0675 (4)	0.7078 (5)	0.4665 (3)	0.0584 (12)	
H15A	−0.1545	0.7427	0.4596	0.070*	
C16	0.0030 (5)	0.7007 (4)	0.5478 (3)	0.0575 (12)	
H16A	−0.0358	0.7327	0.5959	0.069*	
C17	−0.2036 (5)	0.7208 (5)	0.2988 (3)	0.0742 (15)	
H17A	−0.2363	0.7163	0.2372	0.111*	
H17B	−0.2020	0.8094	0.3177	0.111*	
H17C	−0.2624	0.6718	0.3319	0.111*	

### Atomic displacement parameters (Å^2^)

**Table:**

	*U*^11^	*U*^22^	*U*^33^	*U*^12^	*U*^13^	*U*^23^
Br1	0.0684 (3)	0.0498 (3)	0.0762 (4)	0.0041 (2)	0.0140 (2)	−0.0208 (2)
Br2	0.0810 (4)	0.0889 (5)	0.0578 (3)	0.0361 (3)	0.0266 (2)	0.0049 (3)
O1	0.0587 (18)	0.052 (2)	0.0613 (19)	0.0032 (15)	0.0121 (15)	−0.0035 (15)
O2	0.0606 (19)	0.068 (2)	0.067 (2)	0.0150 (17)	0.0113 (15)	−0.0011 (17)
N1	0.069 (2)	0.058 (3)	0.053 (2)	0.000 (2)	0.0149 (18)	−0.0042 (19)
N2	0.068 (3)	0.052 (3)	0.052 (2)	−0.009 (2)	0.0194 (18)	−0.0028 (18)
C1	0.071 (3)	0.072 (4)	0.080 (4)	0.023 (3)	0.010 (3)	0.007 (3)
C2	0.049 (2)	0.038 (3)	0.056 (3)	−0.007 (2)	0.024 (2)	−0.0015 (19)
C3	0.045 (2)	0.037 (3)	0.064 (3)	−0.0049 (19)	0.027 (2)	−0.010 (2)
C4	0.044 (2)	0.042 (3)	0.065 (3)	−0.0026 (19)	0.021 (2)	−0.004 (2)
C5	0.049 (2)	0.046 (3)	0.059 (3)	−0.011 (2)	0.027 (2)	−0.008 (2)
C6	0.061 (3)	0.042 (3)	0.062 (3)	0.000 (2)	0.029 (2)	−0.007 (2)
C7	0.057 (3)	0.047 (3)	0.064 (3)	0.008 (2)	0.026 (2)	0.003 (2)
C9	0.062 (3)	0.051 (3)	0.056 (3)	−0.013 (2)	0.029 (2)	−0.007 (2)
C10	0.069 (3)	0.043 (3)	0.059 (3)	−0.008 (2)	0.025 (2)	−0.007 (2)
C11	0.059 (3)	0.034 (3)	0.055 (3)	−0.002 (2)	0.022 (2)	0.0010 (19)
C12	0.053 (3)	0.036 (3)	0.065 (3)	0.002 (2)	0.022 (2)	0.002 (2)
C13	0.052 (2)	0.037 (3)	0.055 (3)	0.006 (2)	0.019 (2)	0.0032 (19)
C14	0.052 (2)	0.037 (3)	0.060 (3)	−0.002 (2)	0.018 (2)	−0.002 (2)
C15	0.051 (3)	0.046 (3)	0.081 (3)	0.006 (2)	0.024 (2)	−0.001 (2)
C16	0.063 (3)	0.044 (3)	0.070 (3)	0.000 (2)	0.028 (2)	−0.012 (2)
C17	0.065 (3)	0.074 (4)	0.082 (4)	0.019 (3)	0.006 (3)	−0.001 (3)

### Geometric parameters (Å, °)

**Table:**

Br1—C3	1.891 (4)	C6—C7	1.380 (6)
Br2—C13	1.889 (4)	C6—H6A	0.9500
O1—C2	1.359 (5)	C7—H7A	0.9500
O1—C1	1.437 (5)	C9—H9A	0.9500
O2—C14	1.351 (5)	C10—C11	1.456 (6)
O2—C17	1.442 (5)	C10—H10A	0.9500
N1—C9	1.269 (6)	C11—C16	1.383 (6)
N1—N2	1.405 (5)	C11—C12	1.399 (5)
N2—C10	1.259 (6)	C12—C13	1.361 (6)
C1—H1B	0.9800	C12—H12A	0.9500
C1—H1C	0.9800	C13—C14	1.394 (6)
C1—H1D	0.9800	C14—C15	1.392 (6)
C2—C3	1.391 (6)	C15—C16	1.383 (6)
C2—C7	1.393 (6)	C15—H15A	0.9500
C3—C4	1.369 (6)	C16—H16A	0.9500
C4—C5	1.396 (6)	C17—H17A	0.9800
C4—H4A	0.9500	C17—H17B	0.9800
C5—C6	1.387 (6)	C17—H17C	0.9800
C5—C9	1.456 (6)		
			
C2—O1—C1	117.9 (4)	N1—C9—H9A	118.6
C14—O2—C17	117.7 (4)	C5—C9—H9A	118.6
C9—N1—N2	113.3 (4)	N2—C10—C11	122.8 (4)
C10—N2—N1	112.5 (4)	N2—C10—H10A	118.6
O1—C1—H1B	109.5	C11—C10—H10A	118.6
O1—C1—H1C	109.5	C16—C11—C12	118.1 (4)
H1B—C1—H1C	109.5	C16—C11—C10	120.3 (4)
O1—C1—H1D	109.5	C12—C11—C10	121.5 (4)
H1B—C1—H1D	109.5	C13—C12—C11	120.3 (4)
H1C—C1—H1D	109.5	C13—C12—H12A	119.8
O1—C2—C3	117.1 (4)	C11—C12—H12A	119.8
O1—C2—C7	124.3 (4)	C12—C13—C14	122.1 (4)
C3—C2—C7	118.6 (4)	C12—C13—Br2	119.6 (3)
C4—C3—C2	121.1 (4)	C14—C13—Br2	118.4 (3)
C4—C3—Br1	119.2 (3)	O2—C14—C15	124.7 (4)
C2—C3—Br1	119.7 (3)	O2—C14—C13	117.6 (4)
C3—C4—C5	120.5 (4)	C15—C14—C13	117.7 (4)
C3—C4—H4A	119.8	C16—C15—C14	120.3 (4)
C5—C4—H4A	119.8	C16—C15—H15A	119.9
C6—C5—C4	118.5 (4)	C14—C15—H15A	119.9
C6—C5—C9	120.7 (4)	C11—C16—C15	121.5 (4)
C4—C5—C9	120.7 (4)	C11—C16—H16A	119.3
C7—C6—C5	121.0 (4)	C15—C16—H16A	119.3
C7—C6—H6A	119.5	O2—C17—H17A	109.5
C5—C6—H6A	119.5	O2—C17—H17B	109.5
C6—C7—C2	120.2 (4)	H17A—C17—H17B	109.5
C6—C7—H7A	119.9	O2—C17—H17C	109.5
C2—C7—H7A	119.9	H17A—C17—H17C	109.5
N1—C9—C5	122.8 (4)	H17B—C17—H17C	109.5
			
C9—N1—N2—C10	167.5 (4)	N1—N2—C10—C11	174.2 (4)
C1—O1—C2—C3	179.5 (4)	N2—C10—C11—C16	174.6 (4)
C1—O1—C2—C7	−1.6 (6)	N2—C10—C11—C12	−7.6 (7)
O1—C2—C3—C4	179.5 (3)	C16—C11—C12—C13	0.6 (7)
C7—C2—C3—C4	0.5 (6)	C10—C11—C12—C13	−177.3 (4)
O1—C2—C3—Br1	0.3 (5)	C11—C12—C13—C14	−1.1 (7)
C7—C2—C3—Br1	−178.7 (3)	C11—C12—C13—Br2	179.7 (3)
C2—C3—C4—C5	−0.4 (6)	C17—O2—C14—C15	−1.6 (7)
Br1—C3—C4—C5	178.8 (3)	C17—O2—C14—C13	179.0 (4)
C3—C4—C5—C6	0.0 (6)	C12—C13—C14—O2	179.8 (4)
C3—C4—C5—C9	−178.9 (4)	Br2—C13—C14—O2	−1.0 (5)
C4—C5—C6—C7	0.2 (6)	C12—C13—C14—C15	0.4 (7)
C9—C5—C6—C7	179.1 (4)	Br2—C13—C14—C15	179.6 (3)
C5—C6—C7—C2	0.0 (6)	O2—C14—C15—C16	−178.6 (4)
O1—C2—C7—C6	−179.2 (4)	C13—C14—C15—C16	0.8 (7)
C3—C2—C7—C6	−0.4 (6)	C12—C11—C16—C15	0.6 (7)
N2—N1—C9—C5	177.2 (4)	C10—C11—C16—C15	178.4 (4)
C6—C5—C9—N1	171.8 (4)	C14—C15—C16—C11	−1.3 (7)
C4—C5—C9—N1	−9.3 (6)		
